# The Influence of Prenatal DHA Supplementation on Individual Domains of Behavioral Functioning in School-Aged Children: Follow-Up of a Randomized Controlled Trial

**DOI:** 10.3390/nu13092996

**Published:** 2021-08-27

**Authors:** Jacqueline F. Gould, Peter J. Anderson, Lisa N. Yelland, Robert A. Gibson, Maria Makrides

**Affiliations:** 1 Women and Kids, South Australian Health and Medical Research Institute, 72 King William Road, Adelaide 5006, Australia; lisa.yelland@sahmri.com (L.N.Y.); robert.gibson@adelaide.edu.au (R.A.G.); maria.makrides@sahmri.com (M.M.); 2 School of Psychology & Discipline of Paediatrics, Faculty of Health and Medical Sciences, The University of Adelaide, North Terrace, Adelaide 5000, Australia; 3 Turner Institute for Brain and Mental Health, School of Psychological Sciences, Monash University, Melbourne 3800, Australia; peter.j.anderson@monash.edu; 4 Clinical Sciences, Murdoch Children’s Research Institute, Melbourne 3052, Australia; 5 School of Public Health, Faculty of Health and Medical Sciences, The University of Adelaide, North Terrace, Adelaide 5000, Australia; 6 School of Agriculture, Food and Wine, Waite Campus, University of Adelaide, Adelaide 5000, Australia; 7 Discipline of Paediatrics, Faculty of Health and Medical Sciences, The University of Adelaide, North Terrace, Adelaide 5000, Australia

**Keywords:** DHA, RCT, omega-3 fatty acids, supplementation, behavior, behavioral problems, prenatal

## Abstract

Docosahexaenoic acid (DHA) accumulates in the fetal brain during pregnancy and is thought to have a role in supporting neurodevelopment. We conducted a multicenter, double-blind, randomized controlled trial in women with a singleton pregnancy who were <21 weeks’ gestation at trial entry. Women were provided with 800 mg DHA/day or a placebo supplement from trial entry until birth. When children reached seven years of age, we invited parents to complete the Strengths and Difficulties Questionnaire (SDQ), the Behavior Rating Inventory of Executive Function (BRIEF), and the Conners 3rd Edition Attention-Deficit Hyperactivity Disorder (ADHD) Index to assess child behavior and behavioral manifestations of executive dysfunction. There were 543 parent–child pairs (85% of those eligible) that participated in the follow-up. Scores were worse in the DHA group than the placebo group for the BRIEF Global Executive, Behavioral Regulation and Metacognition Indexes, and the Shift, Inhibit, Monitor, Working Memory, and Organization of Materials scales, as well as for the Conners 3 ADHD index, and the SDQ Total Difficulties score, Hyperactivity/Inattention score, and Peer Relationship Problems score. In this healthy, largely term-born sample of children, prenatal DHA supplementation conferred no advantage to childhood behavior, and instead appeared to have an adverse effect on behavioral functioning, as assessed by standardized parental report scales.

## 1. Introduction

The omega-3 long-chain polyunsaturated fatty acid docosahexaenoic acid (DHA, 22:6n − 3) is known to accumulate in the fetal brain in the last trimester of pregnancy [1,2]. DHA is thought to be critical for appropriate neurodevelopment [3,4,5], which occurs at a rapid rate over the last trimester of pregnancy [6]. Both animal models [7,8,9] and observational studies in humans [10,11,12,13,14] have suggested neurodevelopmental benefits for the offspring after ensuring adequate dietary DHA during pregnancy. Numerous randomized controlled trials (RCTs) have attempted to evaluate the effect of providing supplemental DHA to pregnant women on child brain development through performance-based measures such as intelligence quotient tests. Multiple reviews of these RCTs have concluded very little evidence of an effect of a DHA intervention on cognition [15,16,17,18], executive functioning (higher-order cognitive abilities) [15,18,19], motor [15,16,17,18], or language abilities [15,16,17,18,20]; however, a recent review suggested some behavioral effects [21]. 

Behavioral functioning can be broadly dichotomized into internalizing or externalizing behaviors [22]. Internalizing behaviors reflect the internal psychological environment in terms of emotions and thoughts where problems may result in a child being overly shy or anxious, withdrawing socially, having poor self-esteem, or being less interested in social, academic, or recreational activities [23,24]. Internalizing behavior disorders include depression, anxiety, dissociative disorders, and obsessive-compulsive disorder. Externalizing problems are behaviors or actions that are outwardly expressed towards the environment or other individuals, such as hyperactivity and impulsivity, verbal and physical aggression, opposition to authority, inattention, poor inhibition, destruction or theft of others property, and poor temper control [23,24]. Externalizing behavior problems are considered disruptive disorders and include ADHD, and issues with conduct such as oppositional defiant disorder. 

Whilst behavioral functioning has a complex etiology, DHA has been highlighted as potentially influential [25,26,27,28,29,30,31]. Case–control studies have revealed lower DHA status among children diagnosed with autism spectrum disorder [32] and ADHD [33] when compared with controls. Associations between low cord blood DHA status (reflecting DHA exposure in late pregnancy) and increased likelihood of ADHD symptoms (externalizing behavior) were reported in one observational study [34], and were reported contrastingly with internalizing behaviors but not externalizing behaviors in another study [35]. High seafood intake, the predominant source of dietary DHA, during pregnancy was associated with reduced hyperactivity (externalizing behavior) at 9 years of age [10], while low seafood intake was associated with poorer prosocial behavior in children at 8 years of age [11]. 

Despite positive associations detected in observational studies, RCTs of DHA supplementation have not clearly demonstrated benefits of DHA interventions for behavioral functioning [16,21]. One of the largest RCTs of prenatal DHA supplementation included assessments of child behavior at 1.5 [36], 4 [37], and 7 years of age [38] through standardized parent rating scales. At 1.5 years of age, parents completed the Social-Emotional and Adaptive-Behavior scales of the Bayley Scales of Infant and Toddler Development, Third Edition [36]. There was no effect of the DHA intervention on either of the overall scale scores, although there was an unexpected sex by treatment interaction where females in the DHA group had poorer adaptive behavior scores compared with females in the placebo group [36]. When parents rated behavior again at 4 years of age on the Strengths and Difficulties Questionnaire (SDQ) and the Behavior Rating Inventory of Executive Function (BRIEF), Preschool Edition, children in the DHA group were reported to exhibit more hyperactivity and inattention (symptoms of externalizing behaviors) as well as worse emergent metacognition and ability to plan and organize compared with children in the placebo group [37]. Scores reflecting internalizing behaviors did not differ between the randomization groups [37]. When the children reached 7 years of age, parents again completed the SDQ and BRIEF as well as a measure of symptoms of ADHD (indicating poor externalizing behavior). 

Given that DHA is thought to be beneficial for brain development in general [10,11,12,13,14], alongside suggestions that DHA supplementation may reduce externalizing behavioral problems such as ADHD [29,39] or oppositional behaviors [40], it is important to further explore the unexpected effects on behavior and whether there are effects on global behavior or specific behavioral domains. The assessments of behavior in this large prenatal DHA RCT offer an ideal opportunity to explore the effect of the intervention on numerous domains of behavioral development, as well as the severity of any effect. By 7 years of age, behavioral functioning is reasonably stable [22,41] and, if problematic, may start to adversely affect schooling [42,43,44,45,46,47], and health-related quality of life [48], as well as being predictive of employment and income in adulthood [49]. Only overall summary scores were reported from this study, all of which revealed more behavioral symptoms in the DHA group compared with the placebo group [38]. Summary scores reflect overall functioning (combining internalizing and externalizing behaviors), and do not indicate whether effects are limited to a specific domain [50]. To better understand the pattern of behavioral problems related to DHA supplementation, analysis of the individual clinical scales is necessary. Furthermore, as only mean scores were compared between randomization groups, it is impossible to determine whether there was an increased risk of children being categorized as possibly having dysfunctional behavior requiring follow-up. The objective of the current study was to determine the effect of prenatal DHA supplementation on the individual scales of the behavioral assessments completed at 7 years of age, particularly measures of externalizing behavior, in addition to whether any differences resulted in behavioral symptoms that could be considered clinically dysfunctional. Based on the behavioral assessments in this cohort at 1.5 [36] and 4 [37] years of age, this study explored the hypothesis that effects of DHA would be restricted to externalizing behaviors. 

## 2. Materials and Methods

### 2.1. Participants 

Methods and results for the DHA to Optimize Maternal Infant Outcome (DOMInO) trial and follow-up studies are published [36,38,51,52]. To summarize, women were eligible to participate in this multicenter, parallel, double-blind, randomized controlled trial if they had a singleton pregnancy less than 21 weeks’ gestation. Exclusion criteria were current use of a DHA supplement or anticoagulant therapy, bleeding disorders (contraindicating fish oil consumption), history of drug or alcohol abuse, current participation in another fatty acid trial, inability to provide written informed consent, known major fetal abnormality, and any language but English being the main language spoken at home. A computer-driven telephone randomization service employing an independently generated randomization schedule was used to allocate women to receive three capsules of either DHA-rich fish oil (800 mg DHA/day and 100 mg/d eicosapentaenoic acid; Incromega 500 TG, Croda Chemicals, East Yorkshire, England) or vegetable oil daily from trial entry until delivery [36]. The randomization schedule used balanced variable-sized blocks with stratification by enrolment (medical) center and parity (first birth vs. subsequent birth) to ensure approximately equal numbers in each randomization group. All investigators and study staff were blinded to group allocation throughout the trial and follow-ups. Women could request knowledge of their group allocation from an independent statistician once the primary outcome analyses from the 1.5-year assessments were complete, although the majority (92%) remained blinded throughout the follow-up studies to 7 years of age. Women who requested to know their group allocation were instructed to keep this knowledge confidential, and not to discuss this with study staff. 

A total of 2399 women from five hospitals were recruited to the trial. There were 726 mother–child pairs (DHA group *n* = 351, placebo *n* = 375) from two hospitals selected a priori for long-term neurodevelopmental follow-up, including all children born preterm (*n* = 96) and a random selection of children born full-term (*n* = 630) [36]. By the 7-year follow-up, there were 638 mother–child pairs (DHA group *n* = 310, placebo *n* = 328) remaining in the study (who had not previously withdrawn from the DOMInO trial or died). Families were invited to the follow-up prior to the child’s seventh birthday. Consenting families attended an appointment at a hospital clinic room (at the Women’s and Children’s Hospital or the Flinders Medical Centre in Adelaide, South Australia) or a local community center (such as a library or school) and were assessed with a battery of developmental assessments and parents completed questionnaires [38,51]. If this was not possible, children were assessed at home. 

Parents completed a hard (paper) copy of each behavioral questionnaire whilst their child was undergoing the assessments in a different room. If preferred, parents could complete the questionnaires through interview with study staff at the appointment, or over the phone. The results of the developmental assessments, as well as the overall summary scores from the parent-completed questionnaires have been previously published [38]. 

All procedures were approved by an overseeing Human Research Ethics Committee. The original trial was reviewed and approved by the ethics committee for the Children, Youth, and Women’s Health Service (approval code REC1657/12/2007, approved 6 September 2005) and the 7-year follow-up was reviewed and approved by the committee of the Women’s and Children’s Health Network (approval code REC2526/12/15;HREC/12/WCHN/112, approved 4 February 2013). Mothers provided written informed consent prior to enrolment in the DOMInO Trial, and a legal guardian provided written informed consent on behalf of the child for the 7-year assessments. Pregnant women were originally enrolled into the DOMInO trial between October 2005 and January 2008, and the 7-year assessments were completed between March 2013 and August 2015. 

### 2.2. Strengths and Difficulties Questionnaire (SDQ) 

The SDQ is a 25-item report of symptoms of behavioral problems as well as strengths, with parents rating each item on a -point scale [53]. There are five scales, four of which assess problematic behaviors. The Conduct Problems scale assesses behaviors that indicate a child does not conform to societal behavioral expectations or respect the rights of others, such as disobedience, fighting with other children, lying, and stealing. The Hyperactivity/Inattention scale reflects symptoms of ADHD, for example distractibility, excessive fidgeting, poor concentration and attention span, impulsivity, and restlessness. The Emotional Symptoms scale captures mood and emotional reactions disproportionate to the stimulus, such as fearfulness, anxiousness, low confidence, and unhappiness. The Peer Relationship Problems scale assesses the ability to form relationships with other children their age, such as having friends their own age, being liked by other children, and preferring to play with other children rather than solitary play. The fifth scale, the Prosocial Behavior scale, measures strengths in terms of voluntary consideration of others, for example helpfulness, kindness, and thoughtful actions towards others.

The Conduct Problems and Hyperactivity/Inattention scales reflect externalizing behaviors, whilst the Emotional Symptoms and Peer Relationship Problems scales indicate internalizing behavioral problems [54]. The four behavior problem scales are summarized in an overall Total Difficulties Score [53]. An Impact score also captures whether behavioral difficulties are perceived to have an adverse impact on the child or their family. 

SDQ scores are not age standardized and higher scores on any scale indicate more perceived behavioral problems, with the exception of the Prosocial Behavior scale, where higher scores indicate more strengths in this area [53]. A Total Difficulties Score >16 is considered abnormal and warrants further clinical investigation for a possible behavioral problem. Among the scales, scores are categorized as abnormal when they are >4 for Emotional Symptoms, >3 for Conduct Problems, >6 for Hyperactivity/Inattention, >3 for Peer Problems, and <5 for Prosocial Behavior. A score > 1 on the Impact scale is also considered to be elevated.

### 2.3. Conners 3rd Edition ADHD (Diagnostic and Statistical Manual of Mental Disorders Version IV) Index (Conners 3^TM^ AI-Parent) 

The Conners 3^TM^ AI-parent is a questionnaire capturing symptoms of ADHD, as defined by the Diagnostic and Statistical Manual of Mental Disorders version IV [55]. The Conners 3^TM^ AI-parent is an abbreviated, 10-item version of a larger questionnaire and as such does not contain any subscales that could be explored here. The Conners 3^TM^ AI-parent generates one overall score that is age standardized to a mean of 50 and standard deviation of 10. Higher scores are suggest more symptoms and a score ≥70 (in the very elevated range) is indicative of possible ADHD. 

### 2.4. Behavior Rating Inventory of Executive Function (BRIEF) 

The BRIEF is an 86-item report of behavioral manifestations of executive dysfunction [56]. Parents rate each item on a three-point scale and higher scores indicate more perceived problems. Scores are normed for age to a mean of 50 and standard deviation of 10. Higher scores reflect poorer perceived behavior and scores ≥ 65 are considered indicative of a dysfunction.

The BRIEF generates an overall Global Executive Composite score, as well as the Behavioral Regulation Index (BRI) and Metacognition Index (MI). These two indices are composed of several individual scale scores capturing individual aspects of functioning [56]. 

The BRI reflects the ability to self-monitor and regulate behavior and emotion appropriately, and consists of the Inhibit, Shift, and Emotional Control scales. The Inhibit scale assesses being able to inhibit impulses, reactions, or inappropriate behavior and resist distractions. The Shift scale captures mental flexibility, or the ability to switch between thinking about two different concepts, situations, or tasks. The Emotional Control scale refers to the ability to regulate one’s own emotions and emotional responses. 

The MI captures the ability to control and regulate conscious cognitive processes, such as sustaining ideas and tasks in working memory as well as planning and organizing problem-solving approaches, and consists of the Initiate, Working Memory, Plan/Organize, Monitor, and Organization of Materials scales. The Initiate scale reflects an individual’s ability to commence a task or instigate a response or problem-solving strategy independently. The Working Memory scale measures representational memory, or the capacity to hold key pieces of information in mind, possibly involving some encoding of the information, in order to achieve a specific short-term goal. The Plan/Organize scale captures the ability to anticipate, plan, and manage current and future-oriented tasks such as organizing key information and ideas and developing the appropriate sequential steps needed to accomplish a task. The Monitor scale assesses the ability to monitor and check one’s own work or performance whilst undertaking a task or shortly afterwards, such as to improve accuracy. The Organization of Materials scale is the ability to organize materials in an orderly fashion. 

### 2.5. Statistical Methods 

A sample size of *n* = 726 was originally selected for neurodevelopmental follow-up based on detecting differences between treatment groups in the mean Cognitive Scale of the Bayley Scales of Infant and Toddler Development, Third Edition, at 1.5 years [36]. At 7 years, this sample size provides >80% power to detect a 0.25 SD difference (a small effect size) between the treatment groups in the mean SDQ subscale scores, assuming 75% follow-up.

All analyses were performed on an intention-to-treat basis according to the mother’s allocation to the treatment or placebo group. Analyses were performed according to the pre-specified statistical analysis plan using SAS Version 9.3 and Stata Release 13. Data collected on participants up to the point of withdrawal were included in the analyses. Multiple imputation was performed separately by treatment group using chained equations to create 100 complete datasets for analysis, under the assumption that data were missing at random [57]. The primary analysis was based on imputed data and included all participants who consented to the follow-up study. Secondary analyses were performed on the available data and on imputed data for the 726 children in the original sub-sample, excluding four deaths. All analyses produced similar results and only the results of the primary analysis are presented.

Continuous behavior scores were analyzed using linear regression models, with treatment effects expressed as adjusted mean differences (AMDs; DHA-placebo). Binary outcomes (defined as scores in the normal range versus scores indicative of a possible problem) were analyzed using log binomial regression models, with treatment effects expressed as adjusted relative risks (ARRs; DHA/placebo).

Analyses took into account both the sampling design and probability weights, calculated as the inverse of the probability of selection. A priori secondary analyses were performed to estimate treatment effects separately by infant sex and test for evidence of effect modification by sex, due to the previously reported sex differences in treatment effects on and language at 18 months in this cohort, and results from other studies suggesting that boys and girls may respond differently to supplementation [36,58].

Both unadjusted and adjusted analyses were performed, with adjustment for the stratification variables (center and parity), as well as child sex and mother’s secondary education, further education, and smoking status at baseline. Unadjusted and adjusted analyses produced similar results, and both are presented in the results tables, but only the adjusted analyses are reported or interpreted in the text. Statistical significance was assessed at the two-sided *p* < 0.05 level. All outcomes presented are secondary and no adjustment was made for multiple comparisons; hence, the results of these analyses should be interpreted with caution. 

Post-randomization child demographics and clinical characteristics were compared between treatment groups based on the available data using t-tests for continuous variables, and chi-squared tests for categorical variables, accounting for the sampling design and weights.

## 3. Results

Of the 726 infants selected a priori for follow-up, there were 638 eligible and approached for the 7-year follow-up. There were 95 families that did not consent to the follow-up (DHA group *n* = 51, placebo *n* = 44), 52 of which were not able to be located and contacted, and 43 declined the follow-up with the predominant reason being that they were too busy to participate. A total of 543 families consented to the assessment at 7 years (75% of the 726 originally selected for neurodevelopment follow-up and 85% of the 638 invited). Participant characteristics in the subset consenting to the follow-up were comparable between randomized groups at baseline and at 7 years (Table 1), with the exception of maternal smoking, which was accounted for in analyses. There were similar proportions of females and males in each randomization group (DHA group females *n* = 133, 51.4%; placebo group females *n* = 140, 49.3%).

### 3.1. Strengths and Difficulties Questionnaire (SDQ) 

As previously reported, the Total Difficulties score of the SDQ was 1.09 points higher on average in the DHA group compared with the placebo group (*p* = 0.02, Table 2) [38]. This appeared to be driven by effects within the Hyperactivity/Inattention (AMD 0.49, 95% CI 0.13 to 0.85; *p* = 0.01) and Peer Relationship Problems (AMD 0.27, 95% CI 0.01 to 0.53; *p* = 0.04) subscales. Children in the DHA group were more likely to be categorized as being in the at-risk range on the SDQ Hyperactivity subscale (Table 3; ARR 1.56, 95% CI 1.12 to 2.17; *p* = 0.01) and to have an Impact score indicative of a possible behavior problem that was substantially adversely impacting their everyday functioning (ARR 1.44, 95% CI 1.07 to 1.94; *p* = 0.02) compared with the placebo group. There was evidence of a sex by treatment interaction on the Hyperactivity/Inattention mean score (AMD 0.97, 95% CI 0.43 to 1.51; *p* < 0.01, interaction *p* = 0.01) and the proportion of scores in the abnormal range (ARR 2.27, 95% CI 1.50 to 3.44; *p* = 0.01; interaction *p* = 0.01), where males in the DHA group scored more poorly than males in the placebo group. Males in the DHA group also appeared to have worse scores on the Total Difficulties score, although the interaction tests did not reach statistical significance.

### 3.2. Conners 3rd Edition ADHD Diagnostic and Statistical Manual of Mental Disorders Version IV Index (Conners 3^TM^ AI-Parent) 

As stated in the primary results paper [38], children in the DHA group had scores that were 2.84 points higher on average (reflecting more symptoms) on Conners 3^TM^ AI-parent ADHD index than children from the placebo group (*p* = 0.02). The mean score for children in the DHA group (60.94) fell just within the elevated range (scores 60–69), indicating more concerns about symptoms of ADHD than is typical. However, there was limited evidence of a difference between the randomization groups in the proportion of children who scored in the clinical range (*p* = 0.06). There was evidence of a sex by treatment interaction (*p* = 0.02), where males in the DHA group had higher scores on average than placebo group males (AMD 5.79, 95% CI 2.13 to 9.44; *p* < 0.01) and were more likely to fall within the abnormal range, although the interaction test was not statistically significant (*p* = 0.36).

### 3.3. BRIEF

Parent-reported assessments indicated more perceived problems in the DHA group than the placebo group (Table 2). As previously reported [38], overall scores on the BRIEF and the Global Executive Composite were 2.38 points higher on average in the DHA group (*p* = 0.01). More children in the DHA group also had Global Executive Composite scores in the at-risk range than did children in the placebo group (Table 3; ARR 1.48, 95% CI 1.07 to 2.05; *p* = 0.02). These effects appeared to have been driven by a negative effect of DHA supplementation predominantly within males; however, the sex interaction effects did not reach statistical significance (*p* = 0.08). 

The BRI was 2.09 points higher on average in the DHA group (95% CI 0.40 to 3.79; *p* = 0.02). The Inhibit (AMD 2.40, 95% CI 0.79 to 4.00; *p* < 0.01) and Shift (AMD 2.05, 95% CI 0.33 to 3.78; *p* = 0.02) scales were the only scores to differ between groups and were elevated in the DHA group, and children in the DHA group were more likely to score in the at-risk range for the Inhibit scale (Table 3; ARR 1.49, 95% CI 1.04 to 2.13; *p* = 0.03). Exploratory analyses by sex suggested that there were no effects within females, whereas the DHA group males had elevated (ie. poorer) Shift scale (53.89 vs. 49.81, AMD 3.89, 95% CI 1.37 to 6.41; *p* < 0.01; interaction *p* = 0.04), BRI, and Inhibit scale scores than placebo group males, although the interaction test did not reach statistical significance for the BRI and Inhibit scale.

The MI was 2.25 points higher on average in the DHA group compared with the placebo group (95% CI 0.57 to 3.92; *p* = 0.01). The Working Memory (AMD 1.88, 95% CI 0.20 to 3.57; *p* = 0.03), Plan/Organize (AMD 2.20, 95% CI 0.49 to 3.92; *p* = 0.01), Monitor (AMD 2.99, 95% CI 1.25 to 4.72; *p* < 0.01), and Organization of Materials (AMD 1.80, 95% CI 0.29 to 3.31; *p* = 0.02) scales were all elevated in the DHA group, as was the proportion of children in the at-risk range for the Monitor scale (ARR 1.72, 95% CI 1.17 to 2.51; *p* = 0.01). Although the mean Initiate scale scores did not differ between groups overall, there was evidence of a sex by treatment interaction effect (*p* = 0.03), where males in the DHA group scored 2.75 points higher on average than males in the placebo group (95% CI 0.69 to 4.80; *p* = 0.01). Males in the DHA group also scored more poorly on the Working Memory scale (AMD 3.73, 95% CI 1.19 to 6.28; *p* < 0.01; interaction *p* = 0.03) and were more likely to score in the at-risk range compared with placebo group males (ARR 1.59, 95% CI 1.08 to 2.35; *p* = 0.02; interaction *p* = 0.01). The MI, Plan/Organize scale, and Monitor scale also appeared to be worse in the DHA group males, with a higher proportion having a problematic score than the placebo group males, although the interaction effects did not reach statistical significance.

## 4. Discussion

It has previously been reported that in this large RCT, DHA supplementation in pregnancy resulted in more parent-rated behavioral symptoms and ADHD symptoms, as well as behavioral manifestations of executive dysfunction at 7 years of age [38]. The current in-depth exploration of the behavioral ratings supported the hypothesis that effects of prenatal DHA (800 mg per day) were most prominent in indices reflecting externalizing behaviors. Additionally, DHA group children appeared to be more likely to score in the at-risk range for externalizing behavioral problems more frequently than placebo group children. The poorer scores in the DHA group were largely driven by an effect within males. These results are somewhat consistent with the findings of potentially adverse effects on externalizing behavior at 1.5 [36] and 4 [37] years of age; although, at 1.5 years, adverse effects were detected within females only [36], and at 4 [37] years of age, there was no sex by treatment interaction. Performance-based assessments of executive functioning (administered by study assessors) were largely null at 4 and 7 years of age, with the exception that males in the DHA group had slightly poorer inhibition and mental flexibility compared with males in the placebo group [38], which is consistent with the differences found on the parent-rated Shift and Inhibit scales. Importantly, the effect sizes of the potentially adverse effects of the DHA intervention were relatively small, even though slightly more DHA group children were classified as being in the at-risk range, suggesting the magnitude of an effect is likely negligible. The differences in parent-rated behavior were not reflected in the clinical diagnoses of behavioral problems [38]. It may be that the DOMInO trial has highlighted a true, small underlying adverse effect of DHA on behavior, or these may be chance findings due to the large number of comparisons made. 

The most comparable trial to DOMInO with long-term developmental follow-up is a large RCT of DHA in Australian infants born <33 weeks’ gestation [59]. Supplementation with a high-dose of DHA or a standard-dose of DHA occurred from within a week of birth until infants reached 40 weeks’ gestation (equivalent to full term) [59], which corresponds to the same supplementation period in the DOMInO trial, although supplementation took place ex utero. Parents completed the BRIEF, SDQ, and Conners 3^TM^ AI-parent questionnaires when the children reached 7 years of age [59]. Comparable to DOMInO, behavioral outcomes, particularly externalizing behaviors and executive dysfunction, were slightly worse in infants who received the high-dose DHA; however, the effects appeared to be driven by differences in females [59]. Whilst similar aspects of behavioral functioning were affected by DHA in these large trials, the sex by treatment interaction is unexpectedly inconsistent. It is important to note that in both trials, behavioral functioning was a secondary outcome and may be subject to random error. 

Nine other prenatal DHA RCTs with behavior assessments have reported no positive or negative effects on behavioral functioning [60,61,62,63,64,65,66,67,68,69,70,71]. However, follow-up was typically prior to school age [60,61,62,64,69], samples were small in all but two trials [67,68], none used the measures administered in DOMInO, only two explored a treatment by sex interaction [67,69], and the dose of DHA was generally low and may have been insufficient to elicit an effect [61,62,63,65,66,67,68,69,70,71]. A recent review identified 25 RCTs of DHA supplementation in the first 1000 days that administered a measure of behavior, of which 19 detected no effect of DHA, 5 reported a negative effect, and 1 reported both positive and negative effects of DHA intervention on an aspect of behavior [21]. In a trial of breastfeeding mothers, males in the DHA group had poorer ratings of prosocial behavior compared with males in the placebo group [72]. A separate trial found temperamental indices of externalizing behavior worse in formula-fed infants randomized to DHA compared with infants in the placebo group [73]. When the behavior was measured in a trial conducted in preterm infants, effortful control in early childhood was worse in the DHA group among infants from higher income households when compared with children in the placebo group who were also from higher income households, although children in the DHA group were conversely likely to have fewer symptoms of autism spectrum disorder [74,75]. A subsequent follow-up of these children resulted in no differences in parent-rated behavior [76]. The same investigators conducted a DHA intervention in older children displaying symptoms of autism spectrum disorder and found fewer symptoms after the intervention [77]. A RCT conducted in Australian formula-fed infants reported that the DHA group children had poorer scores for externalizing behavior at 6 years of age than did placebo group children, particularly for oppositional defiant behavior and effects were most prominent within males [78]. As with the DOMInO trial, negative effects were largely limited to externalizing behaviors, but again, these were secondary outcomes in studies conducted in developed countries (Australia, the United States of America, and Denmark) with populations likely to be nutritionally replete.

Although there is evidence for a possible adverse effect of early DHA interventions on externalizing behavior, in all trials, behavior was a secondary outcome, and attrition was often high [21]. No differences have been detected in diagnoses of behavioral problems or the use of medications for behavioral problems [21], suggesting that if there are true adverse effects, they are not having a clinically significant impact on functioning. Importantly, there are well over 100 published DHA intervention trials in the first 1000 days, and less than a quarter have assessed behavior [21]. 

DHA is considered necessary for optimal brain development; however, as with many nutrients, it is possible that there is an upper limit for safe levels of DHA. Children in both randomization groups of DOMInO were doing well overall, with average intelligence quotient close to the expected mean of 100 [38] and very few diagnoses of neurological disorders (such as ADHD or a language disorder) or chronic health conditions, likely because the mothers were from a well-nourished population and the majority of children were born full term [38]. It may be that fetal brain development is protected against insufficient DHA [79,80], but may not be protected against excess DHA. Another recent large prenatal RCT in Australia found that high-dose DHA supplementation protected against early preterm birth in women with low DHA status, but conversely increased the risk of early preterm birth in women whose baseline status was high [81]. A RCT in formula-fed infants tested the effects of three formulas with differing doses of DHA and a placebo formula on a range of cognitive, language, and executive functioning tasks across childhood [82]. Authors consistently found that the two middle-DHA doses performed best, whilst the group receiving the highest dose of DHA and the placebo group performed slightly more poorly by comparison [82]. A recent, large observational study identified an inverted U-shaped relation between omega-3 long-chain polyunsaturated fatty acid concentration mid-pregnancy and total grey and white matter volume in children at 10 years of age [83]. Lower maternal omega-3 concentrations were associated with lower child brain volume, but a ceiling effect may have been present at higher concentrations [83]. When considered together, there is evidence emerging that may suggest a dose effect of DHA, in which an excess may cause harm to some outcomes. However, further research is needed to explore this. 

Whilst the DOMInO trial is the largest and most comprehensive study and has the advantages of a robust trial design and low attrition, with a low risk of bias, there are limitations to consider. Primarily, the results presented here are secondary outcomes and there are a substantial number of group comparisons, meaning we cannot exclude the possibility of random error. The behavioral questionnaires administered are designed as screening tools to identify potential problems and are not diagnostic. Including teacher ratings of child behavior would have been a beneficial addition to the study, although two smaller trials that included both parent- and teacher-rated behavior found null effects on both [78,84]. Women were able to request knowledge of group allocation after the primary outcome analysis was complete. At the 7-year follow-up, 92% remained blinded, and knowledge of the intervention did not appear to influence any of the post-randomization characteristics, such as DHA intake or home environment, so it is unlikely that knowledge influenced parents’ perceptions of their child’s behavior. The DOMInO sample was comparable to women who gave birth in South Australia at the time of study recruitment, with the exception that the proportion of pregnant women who smoked was higher in the DOMInO sample [85,86], which was adjusted for in all analyses. Given the large sample size of DOMInO, and a relatively low rate of attrition even sever years after enrolment, our results are likely generalizable to other populations in developed Western countries. 

Further research to clarify any effects of DHA on behavior and whether an upper limit of DHA exposure is needed. Whilst it would not be ethical to endorse future additional trials of DHA supplementation for the purpose of detecting harm, current DHA intervention trials should conduct measures of behavior, particularly externalizing behaviors. A new, larger, follow-up of a DHA RCT in almost 1000 children born <29 weeks’ gestation is currently underway, with behavioral assessments administered at 5 years of age [87,88]. The results of this follow-up study will be important for confirming any adverse effects of DHA supplementation on behavioral functioning, and for further investigating whether there is a sex by treatment interaction. Likewise, behavioral assessments of children in the recent and largest prenatal DHA trial (involving over 5000 women) [89] would provide invaluable information to address the ongoing uncertainty. 

## 5. Conclusions

An intervention with about 800 mg DHA per day in a large general sample of well-nourished pregnant women resulted in possible adverse effects on externalizing behavior in the children. This finding needs to be confirmed in future, robust research, preferably through the follow-up of existing, large DHA RCTs.

## Figures and Tables

**Table 1:** Treatment group comparison of baseline and post-randomization demographic, social, and clinical characteristics ^1^.

	DHA	Placebo	*p* Value
Characteristics at Study Entry	*n* = 259	*n* = 284	
Mother primiparous, *n* (%)	142 (54.8)	158 (55.6)	
Mother completed secondary education, *n* (%)	171 (66.0)	192 (67.6)	
Mother completed further education, *n* (%) ^2^	175 (67.6)	203 (71.5)	
Non-smoker before and during early pregnancy, *n* (%)	190 (73.4)	187 (65.8)	
Characteristics at 7 Years	*n* = 259	*n* = 284	
Age at assessment, mean (SD) days	2648 (110)	2652 (134)	0.73
Living with both natural parents, *n*/N (%)	188/256 (73.4)	191/283 (67.5)	0.36
Primary language English at child’s home, *n*/N (%)	246/256 (96.1)	271/283 (95.8)	0.85

^1^ Data are presented as mean (SD) with *p*-value based on *t*-tests, or *n* (%) with *p*-values from a chi-square test. Analyses are based on the raw data and account for the sampling design and weights. No statistical tests were performed for baseline characteristics. ^2^ Degree, diploma, certificate, trade.

**Table 2:** Parent-reported outcomes of behavior at 7 years of age ^1^.

Outcome	DHA Weighted Mean (95% CI) *n* = 259	Placebo Weighted Mean (95% CI) *n* = 284	Un-Adjusted Estimate (95% CI)	Un-Adjusted *p*	Un-Adjusted Interaction *p* ^3^	Adjusted Estimate (95% CI) ^2^	Adjusted *p*	Adjusted Interaction *p* ^3^
**BRIEF**								
Global Executive Composite ^4^	54.89 (53.71, 56.07)	52.54 (51.32, 53.76)	2.35 (0.66, 4.04)	**0.01**	0.10	2.38 (0.67, 4.08)	**0.01**	0.08
Females	54.33 (52.87, 55.80)	53.43 (51.66, 55.21)	0.90 (−1.39, 3.20)	0.44		0.86 (−1.44, 3.16)	0.46	
Males	55.49 (53.62, 57.36)	51.69 (49.99, 53.38)	3.80 (1.28, 6.33)	**<0.01**		3.93 (1.43, 6.44)	**<0.01**	
Behavioral Regulation Index ^4^	53.66 (52.49, 54.83)	51.54 (50.31, 52.76)	2.12 (0.43, 3.81)	**0.01**	0.10	2.09 (0.40, 3.79)	**0.02**	0.10
Females	53.44 (51.95, 54.93)	52.76 (51.09, 54.43)	0.68 (−1.55, 2.91)	0.55		0.68 (−1.59, 2.96)	0.56	
Males	53.89 (52.06, 55.73)	50.36 (48.58, 52.15)	3.53 (0.97, 6.08)	**0.01**		3.55 (1.02, 6.07)	**0.01**	
Inhibit scale	53.23 (52.08, 54.39)	50.86 (49.73, 51.99)	2.37 (0.76, 3.98)	**<0.01**	0.39	2.40 (0.79, 4.00)	**<0.01**	0.31
Females	53.02 (51.56, 54.49)	51.37 (49.85, 52.88)	1.66 (−0.45, 3.76)	0.12		1.57 (−0.54, 3.68)	0.15	
Males	53.46 (51.65, 55.28)	50.38 (48.71, 52.05)	3.08 (0.63, 5.54)	**0.01**		3.24 (0.80, 5.69)	**<0.01**	
Shift scale	53.14 (51.89, 54.39)	51.07 (49.88, 52.25)	2.07 (0.36, 3.78)	**0.02**	**0.02**	2.05 (0.33, 3.78)	**0.02**	**0.04**
Females	52.44 (50.77, 54.11)	52.39 (50.72, 54.05)	0.05 (−2.28, 2.39)	0.96		0.27 (−2.13, 2.66)	0.83	
Males	53.89 (52.01, 55.77)	49.81 (48.12, 51.50)	4.08 (1.56, 6.60)	**<0.01**		3.89 (1.37, 6.41)	**<0.01**	
Emotional Control scale	53.48 (52.32, 54.65)	52.48 (51.22, 53.74)	1.01 (−0.71, 2.72)	0.25	0.20	0.95 (−0.78, 2.67)	0.28	0.18
Females	53.40 (51.85, 54.95)	53.55 (51.79, 55.31)	−0.15 (−2.49, 2.19)	0.90		−0.21 (−2.59, 2.16)	0.86	
Males	53.57 (51.80, 55.34)	51.45 (49.64, 53.26)	2.12 (−0.41, 4.66)	0.10		2.14 (−0.36, 4.64)	0.09	
Metacognition Index ^4^	54.68 (53.51, 55.84)	52.49 (51.29, 53.69)	2.19 (0.52, 3.86)	**0.01**	0.15	2.25 (0.57, 3.92)	**0.01**	0.12
Females	54.01 (52.51, 55.50)	53.03 (51.25, 54.81)	0.98 (−1.34, 3.30)	0.41		0.94 (−1.37, 3.24)	0.43	
Males	55.40 (53.59, 57.21)	51.97 (50.35, 53.59)	3.43 (1.01, 5.86)	**0.01**		3.60 (1.19, 6.02)	<0.01	
Initiate scale	53.12 (52.08, 54.17)	52.09 (50.96, 53.22)	1.03 (−0.51, 2.57)	0.19	0.05	1.05 (−0.51, 2.62)	0.19	**0.03**
Females	52.03 (50.55, 53.50)	52.53 (50.75, 54.31)	−0.50 (−2.82, 1.81)	0.67		−0.59 (−2.90, 1.71)	0.62	
Males	54.31 (52.83, 55.78)	51.67 (50.26, 53.08)	2.63 (0.59, 4.68)	**0.01**		2.75 (0.69, 4.80)	**0.01**	
Working Memory scale	54.46 (53.28, 55.65)	52.79 (51.60, 53.98)	1.67 (−0.02, 3.35)	0.05	0.07	1.88 (0.20, 3.57)	**0.03**	0.03
Females	52.67 (51.24, 54.10)	52.50 (50.83, 54.17)	0.17 (−2.03, 2.37)	0.88		0.09 (−2.13, 2.31)	0.94	
Males	56.40 (54.50, 58.31)	53.08 (51.38, 54.78)	3.32 (0.76, 5.89)	**0.01**		3.73 (1.19, 6.28)	**<0.01**	
Plan/Organize scale	55.52 (54.28, 56.76)	53.31 (52.14, 54.48)	2.21 (0.51, 3.91)	**0.01**	0.05	2.20 (0.49, 3.92)	**0.01**	0.06
Females	55.01 (53.52, 56.50)	54.52 (52.80, 56.24)	0.49 (−1.78, 2.75)	0.67		0.56 (−1.71, 2.84)	0.63	
Males	56.07 (54.04, 58.09)	52.15 (50.56, 53.74)	3.91 (1.34, 6.49)	**<0.01**		3.89 (1.30, 6.48)	**<0.01**	
Organization of Materials scale	54.71 (53.60, 55.81)	52.85 (51.85, 53.86)	1.85 (0.35, 3.35)	**0.02**	0.73	1.80 (0.29, 3.31)	**0.02**	0.84
Females	55.37 (53.81, 56.93)	53.28 (51.80, 54.76)	2.09 (−0.07, 4.24)	0.06		1.95 (−0.19, 4.10)	0.07	
Males	54.00 (52.43, 55.57)	52.45 (51.07, 53.82)	1.55 (−0.53, 3.63)	0.14		1.64 (−0.47, 3.75)	0.13	
Monitor scale	52.51 (51.28, 53.73)	49.48 (48.24, 50.72)	3.03 (1.29, 4.77)	**<0.01**	0.37	2.99 (1.25, 4.72)	**<0.01**	0.35
Females	52.42 (50.90, 53.94)	50.20 (48.39, 52.02)	2.21 (−0.14, 4.57)	0.07		2.17 (−0.17, 4.51)	0.07	
Males	52.61 (50.65, 54.56)	48.79 (47.08, 50.50)	3.82 (1.23, 6.41)	**<0.01**		3.83 (1.26, 6.40)	**<0.01**	
**Conners 3^TM^ AI-parent**								
ADHD t score ^4^	60.94 (59.10, 62.78)	58.37 (56.74, 60.00)	2.56 (0.13, 5.00)	**0.04**	**0.04**	2.84 (0.38, 5.30)	**0.02**	**0.02**
Females	59.43 (57.14, 61.73)	59.36 (56.96, 61.76)	0.07 (−3.23, 3.38)	0.97		−0.03 (−3.35, 3.29)	0.99	
Males	62.56 (59.64, 65.48)	57.43 (55.21, 59.64)	5.13 (1.50, 8.77)	**0.01**		5.79 (2.13, 9.44)	**<0.01**	
**SDQ**								
Total Difficulties Score ^4^	9.71 (9.07, 10.35)	8.63 (7.99, 9.28)	1.08 (0.17, 1.98)	**0.02**	0.16	1.09 (0.18, 2.00)	**0.02**	0.14
Females	8.89 (8.11, 9.67)	8.43 (7.56, 9.29)	0.47 (−0.69, 1.62)	0.43		0.42 (−0.75, 1.58)	0.48	
Males	10.60 (9.58, 11.62)	8.83 (7.86, 9.80)	1.76 (0.36, 3.16)	**0.01**		1.79 (0.40, 3.17)	**0.01**	
Emotional Symptoms score	2.21 (2.01, 2.41)	1.97 (1.76, 2.18)	0.24 (−0.05, 0.53)	0.10	0.98	0.23 (−0.07, 0.52)	0.13	0.84
Females	2.38 (2.07, 2.69)	2.14 (1.85, 2.44)	0.24 (−0.19, 0.66)	0.28		0.25 (−0.18, 0.69)	0.25	
Males	2.03 (1.77, 2.28)	1.80 (1.50, 2.10)	0.23 (−0.16, 0.62)	0.25		0.20 (−0.19, 0.58)	0.32	
Conduct Problems score	1.74 (1.56, 1.92)	1.63 (1.45, 1.81)	0.11 (−0.14, 0.36)	0.39	0.21	0.10 (−0.15, 0.36)	0.43	0.17
Females	1.55 (1.33, 1.77)	1.59 (1.35, 1.84)	−0.04 (−0.37, 0.28)	0.79		−0.08 (−0.41, 0.26)	0.65	
Males	1.94 (1.65, 2.24)	1.66 (1.40, 1.92)	0.28 (−0.11, 0.67)	0.16		0.29 (−0.10, 0.68)	0.15	
Hyperactivity score	4.13 (3.87, 4.39)	3.67 (3.42, 3.92)	0.45 (0.09, 0.82)	**0.01**	**0.02**	0.49 (0.13, 0.85)	**0.01**	**0.01**
Females	3.58 (3.27, 3.89)	3.52 (3.16, 3.88)	0.06 (−0.42, 0.54)	0.80		0.03 (−0.45, 0.51)	0.91	
Males	4.71 (4.29, 5.13)	3.82 (3.47, 4.17)	0.90 (0.35, 1.44)	**<0.01**		0.97 (0.43, 1.51)	**<0.01**	
Peer Problems score	1.61 (1.41, 1.80)	1.34 (1.16, 1.52)	0.27 (0.01, 0.53)	0.05	0.65	0.27 (0.01, 0.53)	**0.04**	0.75
Females	1.38 (1.15, 1.61)	1.16 (0.94, 1.38)	0.22 (−0.10, 0.54)	0.18		0.23 (−0.09, 0.54)	0.17	
Males	1.85 (1.54, 2.17)	1.51 (1.23, 1.79)	0.34 (−0.07, 0.76)	0.11		0.31 (−0.10, 0.72)	0.14	
Prosocial Behavior score	8.12 (7.92, 8.32)	8.18 (7.99, 8.38)	−0.06 (−0.34, 0.22)	0.69	0.65	−0.05 (−0.32, 0.23)	0.74	0.45
Females	8.44 (8.19, 8.69)	8.45 (8.19, 8.71)	−0.01 (−0.37, 0.35)	0.95		0.06 (−0.30, 0.42)	0.75	
Males	7.78 (7.47, 8.09)	7.92 (7.63, 8.21)	−0.14 (−0.56, 0.28)	0.52		−0.16 (−0.58, 0.27)	0.47	
Impact score	0.90 (0.73, 1.08)	0.69 (0.53, 0.85)	0.21 (−0.02, 0.45)	0.08	0.65	0.23 (−0.01, 0.46)	0.06	0.57
Females	0.59 (0.41, 0.78)	0.42 (0.27, 0.57)	0.18 (−0.07, 0.42)	0.15		0.16 (−0.09, 0.41)	0.22	
Males	1.24 (0.94, 1.54)	0.95 (0.68, 1.22)	0.29 (−0.12, 0.69)	0.17		0.30 (−0.10, 0.69)	0.15	

^1^ Data are presented as weighted mean (95% CI) with the effect estimate (95% CI) being the mean difference (DHA-Placebo). Analyses are based on 100 imputed datasets and were performed using linear regression models accounting for the sampling design and weights. ^2^ Adjusted for center, parity, infant sex, mother’s secondary education, mother’s further education, and mother’s smoking status. ^3^ *p*-value for infant sex by treatment group interaction term. ^4^ Data reported previously [38]. BRIEF: Behavior Rating Inventory of Executive Function. Conners 3^TM^ AI-parent: Conners 3rd Edition Attention-Deficit Hyperactivity Disorder Diagnostic and Statistical Manual of Mental Disorders version IV Index. SDQ: Strengths and Difficulties Questionnaire.

**Table 3:** Proportion of children with scores within the clinically significant range for behavior and behavioral manifestation of executive functions at 7 years of age ^1^.

Outcome	DHA Weighted % (95% CI) *n* = 259	Placebo Weighted % (95% CI) *n* = 284	Unadjusted Estimate (95% CI)	Un-Adjusted *p*	Un-Adjusted Interaction *p* ^3^	Adjusted Estimate (95% CI) ^2^	Adjusted *p*	Adjusted Interaction *p* ^3^
**BRIEF**								
Global Executive Composite ≥65	20.62 (16.42, 24.81)	14.24 (10.80, 17.67)	1.45 (1.06, 1.98)	**0.02**	0.24	1.48 (1.07, 2.05)	**0.02**	0.27
Females	19.51 (13.76, 25.27)	16.17 (10.98, 21.36)	1.21 (0.78, 1.86)	0.40		1.25 (0.79, 1.95)	0.34	
Males	21.80 (15.67, 27.94)	12.38 (7.87, 16.89)	1.76 (1.11, 2.79)	**0.02**		1.80 (1.13, 2.85)	**0.01**	
Behavioral Regulation Index ≥65	18.12 (14.10, 22.14)	16.56 (12.83, 20.28)	1.09 (0.80, 1.50)	0.57	0.99	1.11 (0.80, 1.53)	0.54	0.93
Females	16.88 (11.47, 22.28)	15.33 (10.12, 20.54)	1.10 (0.69, 1.75)	0.68		1.12 (0.69, 1.84)	0.64	
Males	19.47 (13.48, 25.45)	17.73 (12.42, 23.04)	1.10 (0.71, 1.69)	0.67		1.09 (0.72, 1.67)	0.68	
Inhibit scale ≥65	17.79 (13.77, 21.81)	11.92 (8.70, 15.15)	1.49 (1.05, 2.12)	**0.03**	0.78	1.49 (1.04, 2.13)	**0.03**	0.68
Females	14.97 (9.80, 20.15)	10.54 (6.06, 15.03)	1.42 (0.82, 2.45)	0.21		1.36 (0.76, 2.44)	0.30	
Males	20.83 (14.63, 27.03)	13.24 (8.60, 17.88)	1.57 (0.99, 2.49)	0.05		1.59 (1.00, 2.52)	0.05	
Shift scale ≥65	13.53 (10.01, 17.05)	14.41 (10.93, 17.88)	0.94 (0.66, 1.34)	0.73	0.19	0.96 (0.67, 1.39)	0.83	0.39
Females	10.99 (6.63, 15.34)	15.01 (10.07, 19.95)	0.73 (0.44, 1.23)	0.24		0.80 (0.46, 1.40)	0.44	
Males	16.27 (10.68, 21.87)	13.83 (8.93, 18.74)	1.18 (0.72, 1.93)	0.52		1.12 (0.68, 1.84)	0.66	
Emotional Control scale ≥65	17.62 (13.69, 21.55)	18.93 (15.06, 22.81)	0.93 (0.69, 1.26)	0.64	0.36	0.94 (0.68, 1.28)	0.69	0.35
Females	15.25 (10.11, 20.40)	18.99 (13.43, 24.56)	0.80 (0.51, 1.26)	0.34		0.80 (0.49, 1.28)	0.35	
Males	20.18 (14.18, 26.18)	18.88 (13.46, 24.29)	1.07 (0.71, 1.62)	0.75		1.08 (0.71, 1.63)	0.72	
Metacognition Index ≥65	19.36 (15.23, 23.49)	15.01 (11.44, 18.57)	1.29 (0.94, 1.77)	0.12	0.12	1.28 (0.93, 1.76)	0.14	0.14
Females	17.18 (11.68, 22.67)	17.12 (11.69, 22.55)	1.00 (0.64, 1.57)	0.99		1.01 (0.64, 1.58)	0.98	
Males	21.72 (15.51, 27.93)	12.99 (8.37, 17.61)	1.67 (1.06, 2.64)	**0.03**		1.63 (1.03, 2.58)	**0.04**	
Initiate scale ≥65	16.29 (12.49, 20.09)	14.18 (10.62, 17.73)	1.15 (0.82, 1.62)	0.43	0.39	1.14 (0.80, 1.63)	0.47	0.38
Females	13.72 (8.82, 18.61)	13.99 (8.92, 19.07)	0.98 (0.59, 1.63)	0.94		0.96 (0.57, 1.62)	0.88	
Males	19.06 (13.21, 24.91)	14.35 (9.39, 19.31)	1.33 (0.84, 2.11)	0.23		1.31 (0.82, 2.11)	0.26	
Working Memory scale ≥65	18.93 (14.89, 22.98)	17.23 (13.52, 20.95)	1.10 (0.81, 1.49)	0.54	**0.01**	1.16 (0.85, 1.58)	0.36	**0.01**
Female	11.31 (6.74, 15.89)	16.69 (11.42, 21.97)	0.68 (0.41, 1.13)	0.14		0.67 (0.40, 1.12)	0.13	
Males	27.15 (20.44, 33.86)	17.75 (12.51, 22.99)	1.53 (1.04, 2.25)	**0.03**		1.59 (1.08, 2.35)	**0.02**	
Plan/Organize scale ≥65	21.13 (16.87, 25.38)	17.42 (13.71, 21.13)	1.21 (0.91, 1.62)	0.19	**0.03**	1.20 (0.90, 1.62)	0.22	0.05
Females	18.81 (13.17, 24.45)	21.31 (15.54, 27.07)	0.88 (0.59, 1.32)	0.55		0.91 (0.61, 1.36)	0.66	
Males	23.63 (17.22, 30.04)	13.69 (9.04, 18.35)	1.73 (1.12, 2.66)	**0.01**		1.67 (1.07, 2.59)	**0.02**	
Organization of Materials scale ≥65	23.47 (19.08, 27.86)	17.09 (13.41, 20.78)	1.37 (1.03, 1.83)	**0.03**	0.69	1.32 (0.98, 1.78)	0.07	0.60
Females	26.86 (20.55, 33.16)	20.74 (15.06, 26.42)	1.29 (0.90, 1.86)	0.16		1.24 (0.86, 1.80)	0.25	
Males	19.83 (13.77, 25.88)	13.60 (8.89, 18.30)	1.46 (0.92, 2.31)	0.11		1.45 (0.92, 2.30)	0.11	
Monitor scale ≥65	17.17 (13.18, 21.16)	10.16 (7.17, 13.14)	1.69 (1.16, 2.46)	**0.01**	0.50	1.72 (1.17, 2.51)	**0.01**	0.52
Females	15.94 (10.57, 21.31)	10.73 (6.31, 15.14)	1.49 (0.87, 2.53)	0.14		1.51 (0.88, 2.60)	0.13	
Males	18.50 (12.56, 24.44)	9.61 (5.56, 13.66)	1.93 (1.14, 3.26)	**0.02**		1.94 (1.14, 3.30)	**0.01**	
**Conners 3^TM^ AI-parent**								
ADHD t score ≥70	31.05 (26.25, 35.85)	25.55 (21.27, 29.83)	1.22 (0.97, 1.52)	0.09	0.43	1.25 (0.99, 1.58)	0.06	0.36
Females	25.69 (19.41, 31.97)	23.27 (17.36, 29.18)	1.10 (0.78, 1.57)	0.58		1.10 (0.76, 1.58)	0.62	
Males	36.84 (29.56, 44.11)	27.74 (21.56, 33.92)	1.33 (0.99, 1.78)	0.06		1.37 (1.01, 1.84)	**0.04**	
**SDQ**								
Total Difficulties score >16	14.33 (10.78, 17.88)	12.02 (8.76, 15.27)	1.19 (0.83, 1.72)	0.35	0.38	1.20 (0.82, 1.74)	0.35	0.42
Females	10.72 (6.51, 14.93)	10.80 (6.34, 15.26)	0.99 (0.56, 1.75)	0.98		0.99 (0.56, 1.77)	0.98	
Males	18.22 (12.45, 24.00)	13.18 (8.45, 17.91)	1.38 (0.86, 2.23)	0.19		1.36 (0.83, 2.20)	0.22	
Emotional Symptoms score >4	13.30 (9.85, 16.75)	13.33 (9.89, 16.76)	1.00 (0.69, 1.44)	0.99	0.56	0.97 (0.66, 1.43)	0.89	0.64
Females	16.65 (11.46, 21.84)	15.49 (10.30, 20.67)	1.08 (0.68, 1.70)	0.76		1.04 (0.65, 1.68)	0.87	
Males	9.68 (5.22, 14.14)	11.25 (6.72, 15.78)	0.86 (0.47, 1.58)	0.62		0.87 (0.46, 1.62)	0.65	
Conduct Problems score >3 ^4^	15.98 (12.19, 19.78)	12.02 (8.84, 15.21)	1.33 (0.93, 1.90)	0.12	0.89	1.34 (0.94, 1.91)	0.11	0.84
Females	11.19 (6.86, 15.51)	8.58 (4.51, 12.64)	1.31 (0.71, 2.40)	0.39		1.27 (0.69, 2.35)	0.44	
Males	21.15 (14.85, 27.46)	15.32 (10.45, 20.20)	1.38 (0.89, 2.13)	0.15		1.37 (0.89, 2.13)	0.15	
Hyperactivity score >6	19.80 (15.71, 23.90)	13.28 (9.93, 16.62)	1.49 (1.08, 2.07)	**0.02**	**0.01**	1.56 (1.12, 2.17)	**0.01**	**<0.01**
Females	10.56 (6.13, 14.98)	13.08 (8.35, 17.82)	0.81 (0.46, 1.41)	0.45		0.77 (0.44, 1.37)	0.38	
Males	29.78 (22.90, 36.66)	13.46 (8.75, 18.18)	2.21 (1.46, 3.36)	**<0.01**		2.27 (1.50, 3.44)	**<0.01**	
Peer Problems score >3	13.74 (10.16, 17.33)	10.76 (7.59, 13.93)	1.28 (0.86, 1.89)	0.22	0.55	1.28 (0.86, 1.89)	0.22	0.69
Females	10.25 (5.86, 14.64)	9.18 (5.07, 13.29)	1.12 (0.60, 2.07)	0.72		1.16 (0.63, 2.13)	0.64	
Males	17.51 (11.78, 23.24)	12.27 (7.49, 17.06)	1.43 (0.86, 2.37)	0.17		1.36 (0.82, 2.27)	0.24	
Prosocial Behavior score <5	3.98 (1.87, 6.09)	4.40 (2.37, 6.43)	0.90 (0.45, 1.82)	0.78	0.58	0.97 (0.48, 1.95)	0.93	0.50
Females	2.86 (0.50, 5.22)	3.97 (1.13, 6.81)	0.72 (0.24, 2.15)	0.56		0.73 (0.25, 2.18)	0.58	
Males	5.19 (1.63, 8.76)	4.81 (1.91, 7.71)	1.08 (0.43, 2.67)	0.87		1.18 (0.50, 2.79)	0.71	
Impact score >1	22.64 (18.27, 27.00)	15.57 (12.02, 19.12)	1.45 (1.08, 1.96)	**0.02**	0.34	1.44 (1.07, 1.94)	**0.02**	0.31
Females	16.71 (11.29, 22.14)	9.12 (5.02, 13.23)	1.83 (1.05, 3.20)	**0.03**		1.84 (1.04, 3.25)	**0.04**	
Males	29.03 (22.17, 35.90)	21.74 (16.07, 27.41)	1.34 (0.94, 1.90)	0.11		1.30 (0.91, 1.85)	0.15	

^1^ Data are presented as weighted percentage (95% CI) with the effect estimate (95% CI) being the relative risk (DHA/placebo). Analyses are based on 100 imputed datasets and were performed using log binomial regression accounting for the sampling design and weights. ^2^ Adjusted for center, parity, infant sex, mother’s secondary education, mother’s further education, and mother’s smoking status. ^3^ *p*-value for infant sex by treatment group interaction terms. ^4^ Analyzed using log Poisson regression as convergence issues were encountered with log binomial regression for some imputed datasets. BRIEF: Behavior Rating Inventory of Executive Function. Conners 3^TM^ AI-parent: Conners 3rd Edition Attention-Deficit Hyperactivity Disorder Diagnostic and Statistical Manual of Mental Disorders version IV Index. SDQ: Strengths and Difficulties Questionnaire.

## Data Availability

The data presented this manuscript may be made available upon request to the authors, and with approval from the governing ethics committee.
